# Three-dimensional models of the cervicovaginal epithelia to study host–microbiome interactions and sexually transmitted infections

**DOI:** 10.1093/femspd/ftac026

**Published:** 2022-08-04

**Authors:** Vonetta L Edwards, Elias McComb, Jason P Gleghorn, Larry Forney, Patrik M Bavoil, Jacques Ravel

**Affiliations:** Institute for Genome Sciences, University of Maryland School of Medicine, Baltimore, MD, United States; Department of Microbiology and Immunology, University of Maryland School of Medicine, Baltimore, MD, United States; Institute for Genome Sciences, University of Maryland School of Medicine, Baltimore, MD, United States; Department of Biomedical Engineering, University of Delaware, Newark, DE, United States; Department of Biological Sciences, University of Idaho, Moscow, ID, United States; Department of Microbiology and Immunology, University of Maryland School of Medicine, Baltimore, MD, United States; Department of Microbial Pathogenesis, University of Maryland, Baltimore, MD, United States; Institute for Genome Sciences, University of Maryland School of Medicine, Baltimore, MD, United States; Department of Microbiology and Immunology, University of Maryland School of Medicine, Baltimore, MD, United States

**Keywords:** 3D model, cervicovaginal epithelium, sexually transmitted infections, microbiome

## Abstract

2D cell culture systems have historically provided controlled, reproducible means to analyze host–pathogen interactions observed in the human reproductive tract. Although inexpensive, straightforward, and requiring a very short time commitment, these models recapitulate neither the functionality of multilayered cell types nor the associated microbiome that occurs in a human. Animal models have commonly been used to recreate the complexity of human infections. However, extensive modifications of animal models are required to recreate interactions that resemble those in the human reproductive tract. 3D cell culture models have emerged as alternative means of reproducing vital elements of human infections at a fraction of the cost of animal models and on a scale that allows for replicative experiments. Here, we describe a new 3D model that utilizes transwells with epithelial cells seeded apically and a basolateral extracellular matrix (ECM)-like layer. The model produced tissues with morphologic and physiological resemblance to human cervical and vaginal epithelia, including mucus levels produced by cervical cells. Infection by *Chlamydia trachomatis* and *Neisseria gonorrhoeae* was demonstrated, as well as the growth of bacterial species observed in the human vaginal microbiota. This enabled controlled mechanistic analyses of the interactions between host cells, the vaginal microbiota, and STI pathogens. Affordable and semi high-throughput 3D models of the cervicovaginal epithelia that are physiologically relevant by sustaining vaginal bacterial colonization, and facilitate studies of chlamydial and gonococcal infections.

## Teaser

The new 3D cervical model (top) produces tissue with modphologic and physiological (mucus production, bottom) resemblance to human cervical epithelium.

## Introduction

Eukaryotic cell culture systems have been a staple of host-pathogenesis research for decades as they provide the means to model *in vivo* interactions in a controlled and reproducible *in vitro* environment. The mainstay of this approach is a flat surface 2D model, where cells are grown as a monolayer on a solid, impervious surface, usually plastic- or glass-treated with polymers that enhance cell adhesion. This method is inexpensive, accommodates many adherent cell types, and imaging of the cells is relatively straightforward (Abbott 2003, Hurley and McCormick 2003, Barrila et al. 2010). However, while 2D models have provided a wealth of information on host–pathogen interactions, they do not faithfully reproduce the physiological complexity of these interactions as they occur within or on the host organism. Notably, 2D cell culture systems may not accurately represent *in vivo* cell morphology, lack true cellular junctional complexes, and fail to account for the effect of differing cell types usually found within the environmental milieu (Schmeichel and Bissell 2003, Barrila et al. 2010). Experimentally, the design of the 2D systems is also limiting, as it does not allow the introduction of an air interface or the incorporation of extracellular matrices (ECM) that produce needed signaling and crosstalk molecules. As such, many of the predictions derived from 2D cell culture models do not hold true when applied to *in vivo* situations, as seen in cervical cancer models (Karolina Zuk et al. 2017), and other pathogen–host models (Drummond et al. 2016, David et al. 2014).

Animal models for multiple diseases and conditions have been developed to overcome these obstacles. These afford the ability to follow a progressing infection in a complex environment that can replicate many properties of the human host, e.g. local physiology and host response, but falls short on many others, e.g. the structural and polymicrobial environments. Additional manipulations and modifications are often required to maximize susceptibility to human-specific infectious agents (Dutow et al. 2016, Herbst-Kralovetz et al. 2016, Lavender et al. 2018, Pal et al. 2018, Llewellyn et al. 2019, Raterman and Jerse 2019). The use of animal models for STI research is further complicated by the need to use animal-adapted pathogens strains, as is the case with *Chlamydia trachomatis*, or alternate species that have coevolved with their host as is the case with *C. caviae* and *C. muridarum* (Rank and Sanders 1992, Zhang et al. 1993, Lutz-Wohlgroth et al. 2006, Shaw et al. 2017). Lastly, animal models are often expensive to develop and maintain. This high cost may limit the number of replicate experiments, and thus exhaustive investigations are not usually undertaken.

3D cell culture models provide a practical, cost-effective alternative to animal models, while also greatly improving the modeling value of 2D culture systems. 3D models can capture many aspects of the native *in vivo* physiology, including cell morphology, organization, and communication that cannot be replicated in typical 2D models. This includes, but is not limited to, the ability to replicate complex tissue interactions, create and maintain intercellular interactions including junctional complexes, facilitate differentiation and polarization, mimic cellular behavior, and integrate the site-specific microbial environment (Schmeichel and Bissell 2003, Hjelm et al. 2010, Harrington et al. 2014, Orabi et al. 2017, Xiao et al. 2017, Zhu et al. 2017, Barrila et al. 2018, Ogawa-Tominaga et al. 2018, Millar-Haskell et al. 2019, Gargus et al. 2020). Over the past decade, various 3D cell culture reproductive tract model systems have been developed. These range from hydrogels (Pyles et al. 2014, Ogawa-Tominaga et al. 2018), and self-assembled organoids (Barrila et al. 2010, Hjelm et al. 2010), to microfluidics “organ-on-a-chip” models (Xiao et al. 2017). Hydrogels are usually placed on a scaffold, and cells can be grown within or on top of the hydrogel, with 7–21 days necessary for full differentiation. These models have been used to analyze bacterial growth patterns, as well as targeted aspects of pathogenicity for multiple pathogens, including ZIKA, HSV, *Chlamydia* spp., *Neisseria gonorrhoeae*, and HIV (Medina-Colorado et al. 2017, Nogueira et al. 2017, Zhu et al. 2017, Amerson-Brown et al. 2018, Gorwood et al. 2019, Heydarian et al. 2019, Imle et al. 2019). Similarly, self-assembled organoids can be grown either on a scaffold (i.e. collagen-coated beads) or scaffold-free, where cells are placed in suspension and self-aggregate to form a more complex structure. Organoid-based models have been used for mechanistic studies of bacterial pathogenesis (Hjelm et al. 2010, Radtke and Herbst-Kralovetz 2012, Laniewski et al. 2017, Orabi et al. 2017, Heydarian et al. 2019, Laniewski and Herbst-Kralovetz 2019). 3D models generally closely mimic infections by multiple pathogens (Pyles et al. 2014, Laniewski et al. 2017, Zhu et al. 2017, Amerson-Brown et al. 2018, Heydarian et al. 2019, Ilhan et al. 2019) as well as environmental parameters (Doerflinger et al. 2014, Herbst-Kralovetz et al. 2016, Medina-Colorado et al. 2017) as they occur *in vivo*.

Whereas advances in hydrogel-based and organoid-based systems can recapitulate the 3D environment and multicellular nature needed to mimic aspects of the *in vivo* context, to an extent, reproducibility is difficult owing to their stochastic cellular organization and/or time needed to establish the model. Organ on-a-chip models can overcome some of these limitations. Microfluidic modules that integrate parameters such as flow, mechanical stress, and the introduction of multiple environmental cues in any orientation around the cell(s) of interest can be developed (Cabodi et al. 2005, Choi et al. 2007, Picollet-D’hahan et al. 2021, Tantengco et al. 2021). This allows for organ-like systems that can be functionally maintained for extended periods of time, thus facilitating more in-depth analysis. (Xiao et al. 2017, Gargus et al. 2020). However, the high cost of set up and maintenance of some of these models may not be feasible for many laboratories interested in studying host–pathogen interactions of the female reproductive tract. Indeed, there have been limited efforts toward developing organ-on-a-chip systems to model reproductive tract infections.

In this study, we developed a 3D transwell cell culture model characterized by morphologically and physiologically differentiated vaginal and cervical epithelial cells that support the growth of bacteria found in the vaginal milieu and enable infection by both *C. trachomatis* (Edwards et al. 2019) and *N. gonorrhoeae*. The transwell polyester membrane provides scaffolding support for the epithelial cells while allowing close proximity to an ECM and fibroblast network. By using the noncancerous, mucin-producing cell line (A2EN; Buckner et al. 2011), the model recapitulates critical aspects of the *in vivo* environment where mucins play a significant role (Eggert-Kruse et al. 2000, Nunn et al. 2015). The relatively low cost and short set-up time required to establish the model enables the testing of multiple replicates in parallel under multiple conditions in a semihigh throughput process. This model may serve as a primer for the future development of more elaborate 3D organ-on-a-chip model systems.

## Methods

Abbreviations and all catalog numbers are listed in the Supplemental Materials.

### Cell culture model: collagen coating

**Figure 1.**
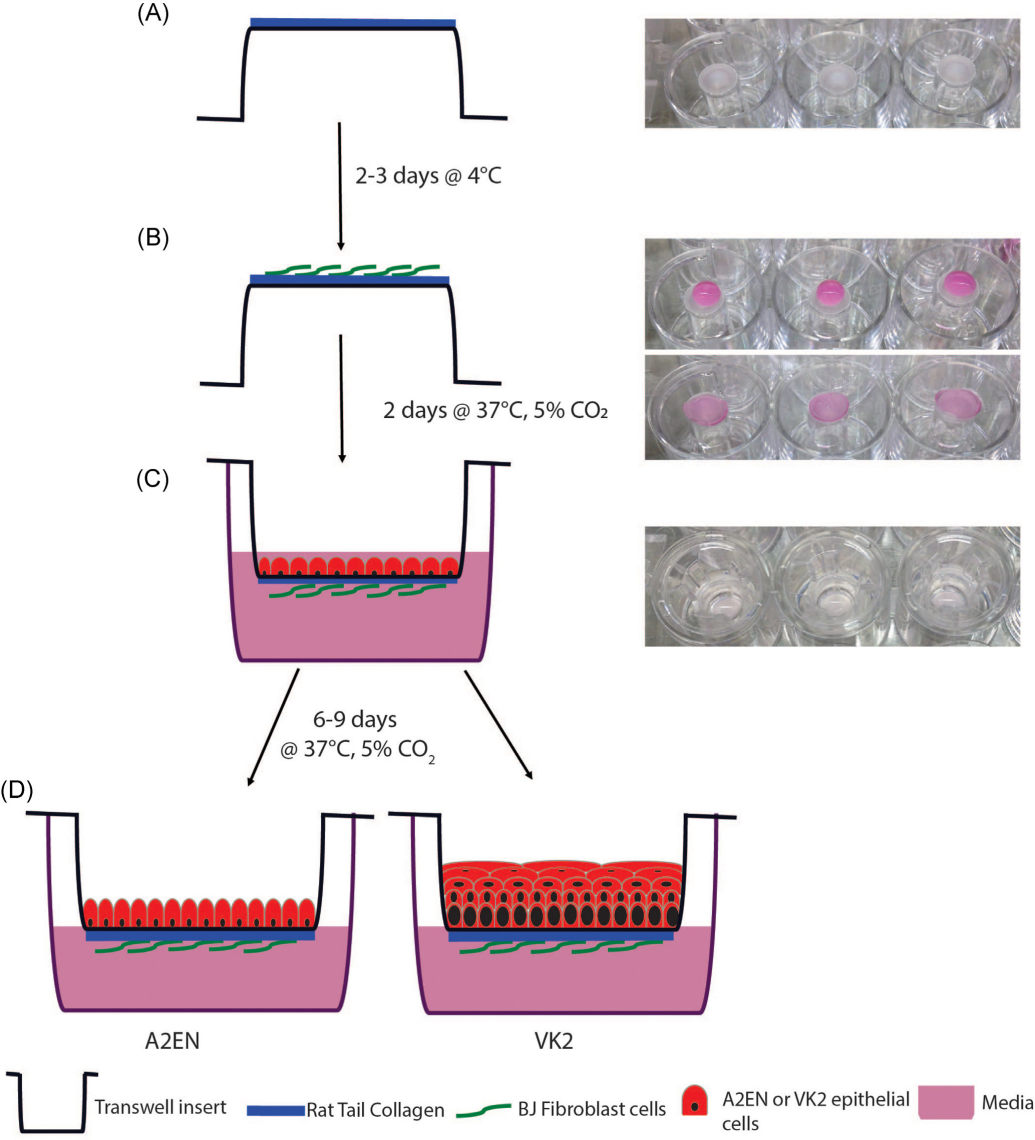
Model set-up. A volume of 70 µl of collagen was added to the basal portion of the inverted transwell **(A)** and stored at 4°C. BJ’s were added to the basal membrane 2–3 days later at 3 × 10^4 ^in a volume of 80–100 µl and incubated at 37°C, 5% CO_2_**(B)**. Epithelial cells (A2EN or VK2) were apically added at 1 × 10^5 ^in a volume of 50–200 µl **(C)**. After 6–9 days of incubation at 37°C, 5% CO_2_ cells were ready to be used in experiments.

Transwell inserts (Corning #3472) were removed from the 24-well plate using glass pipettes or tweezers and placed in an inverted orientation into 12-well plates. To form the collagen coating, all solutions were chilled and placed on ice. A volume of 200 µl 5X RPMI [1:1 mixture of 10X RPMI and tissue culture (TC) water; Sigma #R1145] and 25 µl 1M NaOH (Sigma #S5881) were combined and vortexed thoroughly for 10 s. Rat tail collagen (800 µl; Corning #354236) was added with gentle pipetting to avoid introducing excessive bubbles, and the pH of the mixture was tested. Additional NaOH or RPMI was added in 1 µl or 10 µl increments, respectively, if needed to attain a pH of 6.5 (the final mixture should have a salmon pink color). A total of 70 µl of the collagen mixture was added to the basal surface of each transwell insert, and the plate was covered, ensuring no contact with the collagen surface. The collagen was allowed to gel in a Biosafety Level 2 (BSL2) hood at room temperature for ∼30–60 min. (Fig. 1A). Using clean glass pipettes or tweezers, the inserts were returned to the 24-well plate in the standard orientation and left under the hood for an additional 3 h before being transferred to 4°C for 48–72 h.

### Cell culture model: addition of fibroblasts

After 48–72 h, the transwells were inverted into a 12-well plate using glass pipettes or tweezers. BJ fibroblast cells (ATCC #CRL 2522) at 70%–90% confluency after growth in BJ complete medium [DMEM media (Cellgro #15–013-CV) supplemented with 10% FBS (Sigma #F4135)] were trypsinized using 1 ml of 0.25% Trypsin (Gibco #25200–056) and cell number determined using the Countess Automated Cell Counter. A total of 3 × 10^4^ cells in a volume of 75–80 µl were added to the basal surface of the transwells on top of the collagen (Fig. 1B). The dish was covered and placed in a 37°C, 5% CO_2_ incubator for 6 h. Inserts were then transferred to the 24-well plate in the standard orientation, and BJ complete medium was added (200 μl to the apical compartment and 500 μl to the basal compartment). The transwells were returned to the incubator for an additional ∼42 h.

### Cell culture model: addition of epithelial cells

Either A2EN cervical epithelial cells [kindly provided by Dr Allison Quayle (Buckner et al. 2011)] or VK2/E6/E7 vaginal epithelial cells (ATCC #CRL 2616) were used to make cervical or vaginal models, respectively. A2EN cervical cells were grown in A2EN complete medium [EpiLife media (Gibco #MEPICFPRF) with 100X EDGS supplement (Gibco #S-012–5) and 100X L-glutamine (Lonza #17–605E)], while VK2/E6/E7 vaginal cells (ATCC #CRL 2616) grown in VK2 complete medium [Keratinoctye-SFM (with BPE and EGF; Gibco #10725–018) supplemented with 0.4 M calcium chloride (Amresco #E506) and 100X L-glutamine (Lonza #17–605E)]. Cells were grown until 70%–90% confluent then trypsinized using 1 ml of 0.25% Trypsin. The number of cells was determined using the Countess Automated Cell Counter. BJ complete medium was removed from the transwells, which were then gently rinsed with 500 µl PBS. To seed the epithelial cell layer, using A2EN or VK2 complete medium (cervical and vaginal model respectively), a total of 1 × 10^5 ^epithelial cells in 200 µl of media was added to the apical compartment, and 500 µl of media was added to the basal compartment, and the plate returned to the incubator. After 48 h, the apical and basal media were removed by vacuum aspiration, and fresh medium was added only to the basal compartment to create an epithelial–air interface. Fresh medium (500 µl) was added to the basal compartment every other day. Following culture A2EN: 6 days and VK2: 8 days, epithelial cells were polarized, and no medium could be observed entering the apical compartment from the basal compartment, indicating stable epithelial barrier formation. For the hormone experiment, the model was created as stated above but after the VK2 cells had been seeded for 4 days, 10 ng/ml of β-estradiol was added, and this was maintained until the end of the experiment.

### TEER analysis

The EVOM2 epithelial voltohmmeter (World Precision Instruments) was used as per manufacturer’s instructions. Briefly, since both prongs need to be immersed in medium to facilitate a reading before each reading, culture media were removed and PBS added to the apical (200 µl) and basal (500 µl) compartments. The probe was sterilized by dipping in 70% ethanol, air dried, and then placed in the model with the longer prong resting in the 24-well plate and the shorter prong immersed in the insert (but not resting on the membrane to reduce the probability of accidental puncture). Once the readings stabilized (stopped fluctuating), the resistance value was recorded.

### *Chlamydia trachomatis* infection, microscopy imaging, and cytokine analysis

*Chlamydia trachomatis* serovar L2 (strain LGV/434/Bu) was propagated in HeLa monolayers as previously described in Tan et al. (2010). Briefly, serovar L2 was cultivated in 100 mm^2^ TC dishes containing HeLa cells grown at 37°C, 5% CO_2_. Monolayers were gently rocked for 2 h, fresh medium was added, and the infection was allowed to progress for 48 h. Lysates were harvested, and inclusion-forming units (IFUs) were calculated and stored in sucrose phosphate glutamate (SPG; Tan et al. 2010) at −80°C. Seeds were used directly from −80°C stocks. *Chlamydia trachomatis* was inoculated at a multiplicity of infection (MOI) of 1 or 2.

The A2EN 3D model was inoculated with 2 × 10^5^ IFU *C. trachomatis* in a volume of 50 μl, in the apical compartment and rocked for 2 h at room temperature. Then, the *C. trachomatis* suspension was removed by pipetting, the cells were rinsed with 500 µl PBS, fresh medium (500 µl) added basally, and the model was incubated for an additional 46 h at 37°C, 5% CO_2_.

Following infection, the transwells were prepared for imaging as described in the fluorescence staining section. Images were obtained using a Zeiss Duo 5 confocal microscope, and three consecutive Z stack slices were compressed to create images for confocal analysis. For electronic microscopy, the transwells were placed in glutaraldehyde fixative for processing and imaged on the Tecnai T12 Transmission Electron microscope. For comparative purposes, A2EN cells were grown on coverslips (2D) for 2 days and then infected with *C. trachomatis* at MOI 2 (Reeve et al. 1975). Infection and staining were performed as described below, with images obtained using a Zeiss Axio Imager Z1 (Zeiss). Infected cells were manually identified using the ImageJ software (NIH).

Medium was removed from the basal compartment for cytokine analysis and stored at −80°C. A total of seven cytokines: EGF, IL-6, IL-8, IP10, MDC, PDGF-AA, and RANTES, were analyzed using a Luminex Multianalyte assay at the UMB Cytokine Core Laboratory.

### *Neisseria gonorrhoeae* infection and analysis

3D models containing 2 × 10^5 ^A2EN cells were exposed apically to 100 μl of *N. gonorrhoeae* FA1090 wildtype or *N. gonorrhoeae* Opaless (all *opa* genes deleted) or *N. gonorrhoeae* Δ*pilE*Δ*opa* (*pilE* and all *opa* genes deleted) at MOI of 10 for 6 h at 37°C, 5% CO_2_. The basal medium (500 μl) was then removed, and dilutions were plated on GCK agar plates to determine the number of *N. gonorrhoeae* bacteria that transmigrated within the 6-h incubation period. For comparison, 2 × 10^5 ^HEC-1-B endometrial cells utilizing the same 3D set-up were exposed in parallel, and the transmigrated *N. gonorrhoeae* bacteria were quantified.

### Bacterial growth (*Lactobacillus crispatus* and *Gardnerella vaginalis*) and microscopy imaging

ATCC *Lactobacillus crispatus* (ATCC 33197) strain VPI7635 and *Gardnerella vaginalis* (ATCC 14018) strain 594 were used. The optical densities (OD) of bacterial cultures grown overnight in their respective media (*L. crispatus* in NYCIII and *G. vaginalis* in TSB + 5% horse serum) were determined using an OD to colony-forming units (CFU) conversion of 1 OD represents 1 × 10^9^ CFU. A volume corresponding to 2 × 10^8^ CFUs was added to the experiment culture medium (a 2:1 mixture of complete cell culture medium: bacteria culture medium) to produce a final volume of 1 ml. A 10-fold dilution was then performed using experiment culture medium, and 100 μl was added to the apical compartment of the model (1 × 10^6^ CFU). Cells exposed to bacteria or medium only (no bacteria control) were incubated for 48 h under anaerobic conditions in a 37°C incubator within a Coy chamber. Aliquots of media were removed from the apical compartment and the pH determined using an Apera Instruments PH8500 portable pH meter. Aliquots of 50 μl were used to determine the D(-) and L(+) lactic acid concentrations using the Boehringer Mannheim/R-Biopharm D-Lactic acid/L-Lactic acid kit as per manufacturer’s instructions. Cells were gently rinsed with PBS and either fixed in 2.5% glutaraldehyde (TEM imaging) or 2% PFA (fluorescence imaging). Cells for TEM imaging were provided to the Electron Microscopy Core Imaging Facility for further processing and imaging as described below. Cells for fluorescence *in situ* hybridization (FISH) imaging were processed as described below and imaged on a Zeiss Duo 5 confocal microscope, and five consecutive Z stack slices were compressed to create images. Cells for histology and cell viability imaging were processed as described below and imaged on a Zeiss Duo 5 confocal microscope, where three consecutive Z stack slices were compressed to create images (viability) or a Zeiss Primo Star (histology).

### Hematoxylin and Eosin (H&E) staining

The transwell membrane was excised from the support by rinsing the cell surface with PBS and cutting the perimeter of the membrane with a No. 11 blade on a scalpel. The membrane was placed between two 32 × 25 × 3 mm biopsy pads and secured in a histology cassette. The cassette was immersed in 10% formalin fixative solution for 24 h and then processed by the UMB Pathology Histology Core using SOP NH306. Briefly, slides were placed in hematoxylin, rinsed with water, dipped in acid alcohol, rinsed with water, then sequentially placed in 80% ethanol, eosin, 95% ethanol twice, 100% ethanol twice, and xylene thrice. Mounting media and a coverslip were then added. Resultant slices were imaged at 100X on the Zeiss Primo Star microscope (Zeiss).

### Fluorescence staining

Briefly, cells were rinsed once with 500 µl of Dulbecco’s Phosphate Buffered Saline (PBS), fixed with 4% paraformaldehyde (PFA) for 30 min and permeabilized with 200 μl 0.25% Triton X-100 in PBS for 10 min, followed by treatment with 300 μl 0.1% Triton X-100 in PBS/fish skin gelatin (FSG; 0.66%) for 20 min. The cells were then stained for chlamydial IFUs with 10 μl of 5 μg/ml of mouse antihuman chlamydia LPS (primary Ab; US Biological, MA) in Triton X-100/PBS/FSG solution for 90 min. Secondary antibody staining was done by adding 2 μl of 200 μg/ml of goat antimouse Alexa Fluor 488 in Triton X-100/PBS/FSG solution and incubated 60 min in the dark. Finally, host cells were stained with 2 μl of 500 μg/ml Hoechst in Triton X-100/PBS/FSG solution for 10 min in the dark. Chlamydial inclusions stained green while host cells nuclei stained blue. Cells were imaged using a Zeiss Duo 5 confocal microscope, and three consecutive Z stack slices were compressed to create images for analysis.

### Transmission electron microscopy staining

Cells were rinsed with PBS after removal of medium and fixed in 500 µl of 2% PFA, 2.5% glutaraldehyde, and 0.1 M PIPES buffer (pH 7.4) for at least 1 h. Cells were then washed with 500 µl of 0.1 M PIPES, quenched with 500 µl of 50 mM glycine in 0.1 M PIPES buffer (pH 7) for 15 min, washed and postfixed in 200 µl of 1% (w/v) osmium tetroxide and 0.75% ferrocyanide in 0.1M PIPES buffer at 4°C for 60 min. Following washing, transwell membranes were sliced off the holding cup, stained with 200 µl of 1% (w/v) uranyl acetate in water for 60 min, dehydrated by passage through a graduated ethanol series, and embedded in Spurr’s resin (Electron Microscopy Sciences, Hatfield, PA) following the manufacturer’s recommendations. Resin blocks were trimmed perpendicular to the monolayer grown on the transwell membrane. Ultrathin sections ∼70 nm thickness were cut on a Leica UC6 ultramicrotome (Leica Microsystems, Inc., Bannockburn, IL) and collected onto formva film-coated SynapTek NOTCH-DOT grids (Electron Microscopy Sciences) and examined in a Tecnai T12 transmission electron microscope (Thermo Fisher Scientific, formerly FEI. Co., Hillsboro, OR) operated at 80 keV. Digital images were acquired using a bottom mount CCD camera and AMT600 software (Advanced Microscopy Techniques, Corp, Woburn, MA).

### FISH staining

Cells were stained using a protocol modified from Meaburn (2010). Briefly, cells were rinsed and fixed overnight at 4°C with 2% PFA, then incubated in 200 μl of 0.5% saponin/0.5% Triton X100/PBS mixture for 40 min. This was followed by the addition of 200 μl of 1 N HCL for 20 min, 2X SCC for 10 min, and 50% formamide/2X SCC for 30 min incubations. Cells were then incubated in 300 μl of the hybridization mix containing the FISH probe EUB338-ATT0 for 10 min at 85°C then overnight in a humidity box at 37°C. Next, cells were washed with 500 μl of multiple buffers (a) 50% formamide/2X SSC buffer at 45°C, (b) 1X SSC buffer at 45°C, and (c) 0.05% Tween-20 in 4X SSC buffer at room temperature. Cells were then incubated with 300 μl of Hoechst at 1:500 for 10 min and mounted for imaging. Cells were imaged on the Zeiss Duo 5 microscope (Zeiss) using the 63X objective with 488 and 546 filters.

### Viability staining

At 48 h postinfection, cells were incubated with 300 μl of 4 μM Calcein-AM and 2 μM EthD-III from the Viability/Cytotoxicity Assay Kit for Animal Live and Dead cells (Biotium 30002-T) for 45 min at room temperature as per manufacturer’s recommendations. Cells were imaged at 40X using the 488 and 543 nm excitation wavelengths on the Zeiss Duo 5 microscope (Zeiss). A total of three Z stack slices were overlaid to create a 3D image.

### Statistical analysis

Graphs and statistical analyses were created and performed using the GraphPad Prism Software. When multiple experiments were conducted, or multiple readings taken, error bars represent Standard Deviation (SD). Students *t-*tests were used to statistically analyze individual comparisons. *P*-values: «≤ .05; ««≤ .01; «««≤ .001; and ««««≤ .0001.

## Results

### The epithelial 3D transwell model structurally resembles *in vivo* cervical and vaginal epithelium

**Figure 2.**
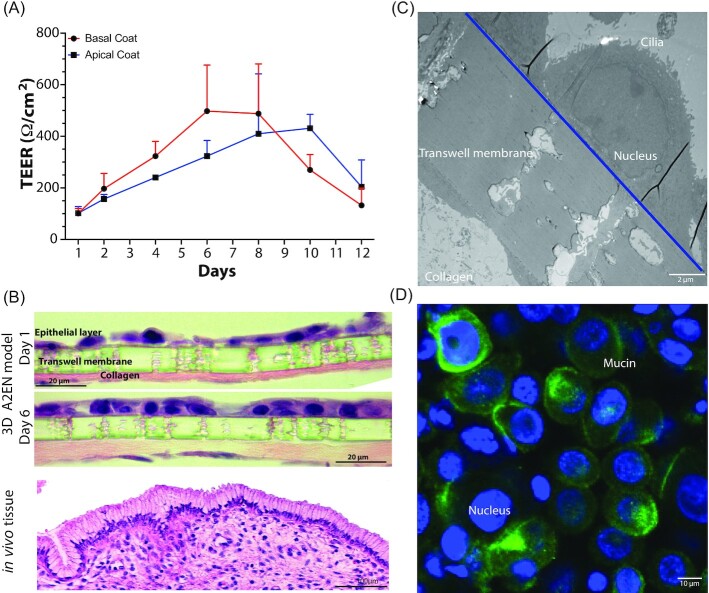
Characterization of the 3D cervical epithelium model (A2EN). Transepithelial resistance values over the course of A2EN epithelial cell transwell 3D model set up **(A)**. Histology (H&E) imaging **(B)** and electron microscopy (TEM) imaging **(C)** of the epithelial cells of the model 6 days post set-up. Confocal imaging of mucin gel formation (MUC-5B) on the model 6 days post set-up **(D)**. Error bars represent SD. Image of *invivo* tissue purchased from shutterstock.com.

**Figure 3.**
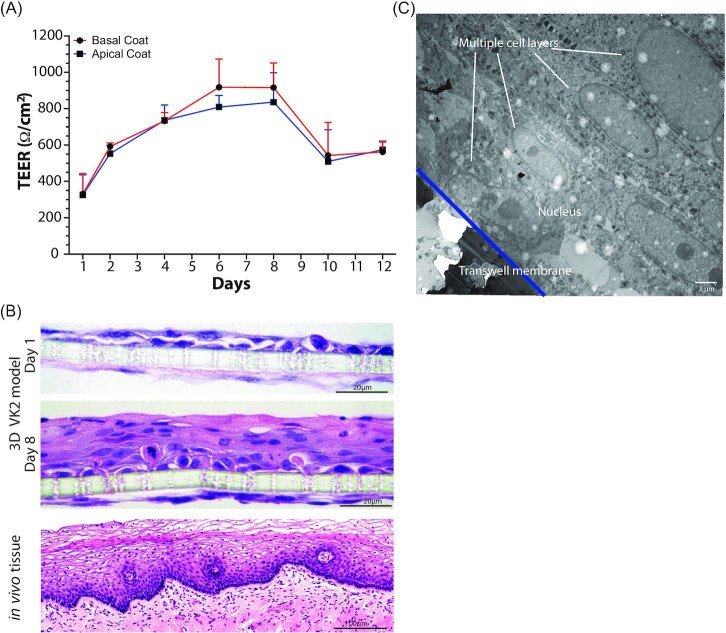
Characterization of the 3D vaginal epithelium model (VK2). Transepithelial resistance values over the course of VK2 epithelial cell transwell 3D model set up **(A)**. Histology (H&E) imaging **(B)** and electron microscopy (TEM) imaging **(C)** of the epithelial cells of the model 8 days post set-up. Error bars represent SD. Image of *invivo* tissue purchased from shutterstock.com.

Transwells, basally coated with collagen and in the presence of human fibroblasts mimicking the *in vivo* basement membrane of the epithelium, were used to develop a 3D model of the female reproductive tract epithelia (Fig. 1). In these models, basal only feeding and air interface exposure afforded the establishment of vaginal and cervical epithelia that morphologically closely resemble the structure of these epithelia *in vivo* (Figs 2 and 3). Polarization of epithelial cells over time is usually an indication of their stage of development. We initially tested multiple types of collagen and coating methods to determine optimal conditions. Epithelial barrier integrity was evaluated using TEER values (Powell 1981) measured over time as the epithelial tissue formed. Basal collagen coating and fibroblast embedding showed a gradual increase in TEER values with peaks of approximately 500 ohms/cm^2^ on day 6 for A2EN cervical epithelial cells (Fig. 2A) and 900 ohms/cm^2^ on day 8 for VK2 vaginal epithelial cells (Fig. 3A). Other collagen coating and fibroblast embedding methods were tested, including apical coating (Figs 2A and 3A) and basal or apical coating with embedded fibroblasts (data not shown). Embedding of fibroblasts was detrimental to the integrity of the collagen layer and caused delamination from the transwell membrane.

Histology and electron microscopy techniques were used to evaluate the structural and morphological features of the two epithelial models. Hematoxylin and eosin (H&E) staining confirmed the increased cell density and polarization of A2EN cervical epithelial cells (Fig. 2B) on day 6 as compared to day 1. Morphologically, the epithelial structure was similar to that observed in histological images of cervical tissue, which comprises a compact single layer of epithelial cells (Fig. 2B). Measuring the thickness of these cells showed that there was a 25% decrease in thickness (∼6 µm) of the epithelial layer in the absence of collagen and fibroblasts (Figure S2, Supporting Information). Transmission electron microscopy (TEM) further confirmed these observations. The A2EN cervical epithelial cells form a monolayer of cells in tight contact with each other. An intact nucleus and cilia on the cell’s surface were also observed (Fig. 2C).

An essential feature of cervical cells is their ability to produce mucus (Adnane et al. 2018, Han et al. 2020). Therefore, A2EN cervical epithelial cells were selected for their demonstrated ability to produce mucus, a unique and important feature of this cell line (Buckner et al. 2011). Furthermore, immunostaining for mucin 5B, a major protein component of mucus, shows that mucus is produced over a significant portion of the apical surface of the A2EN cervical epithelium (Fig. 2D), thus recapitulating a critical functional property of the cervical epithelium (Han et al. 2020).

Histology and electron microscopy imaging of the VK2 vaginal epithelial model revealed a pronounced stratification at day 8 as compared to day 1 with multiple layers (up to seven) of cells observed (Fig. 3B and C). There was also a 24% reduction (∼12 µm) in thickness of the epithelial layer in the absence of collagen and fibroblasts (Figure S2, Supporting Information). Further, maturation of VK2 epithelial cells was observed, with more mature cells in the upper layers and more immature cells in the lower layers. This organization mimics an integral feature of the vaginal epithelium, as glycogen, a key metabolite supporting the growth of the vaginal microbiota, accumulates in mature epithelial cells (Anderson et al. 2014). Hormones play a significant role in modulating the epithelium of the female reproductive tract. In our vaginal (VK2) model, the addition of estrogen resulted in the formation of additional layers of epithelial cells and the thickness of the epithelium increased by 30% (∼12.5 µm; Figure S2, Supporting Information).

### 3D cervical A2EN cells are infected by *C. trachomatis* and *N. gonorrhoeae*

**Figure 4.**
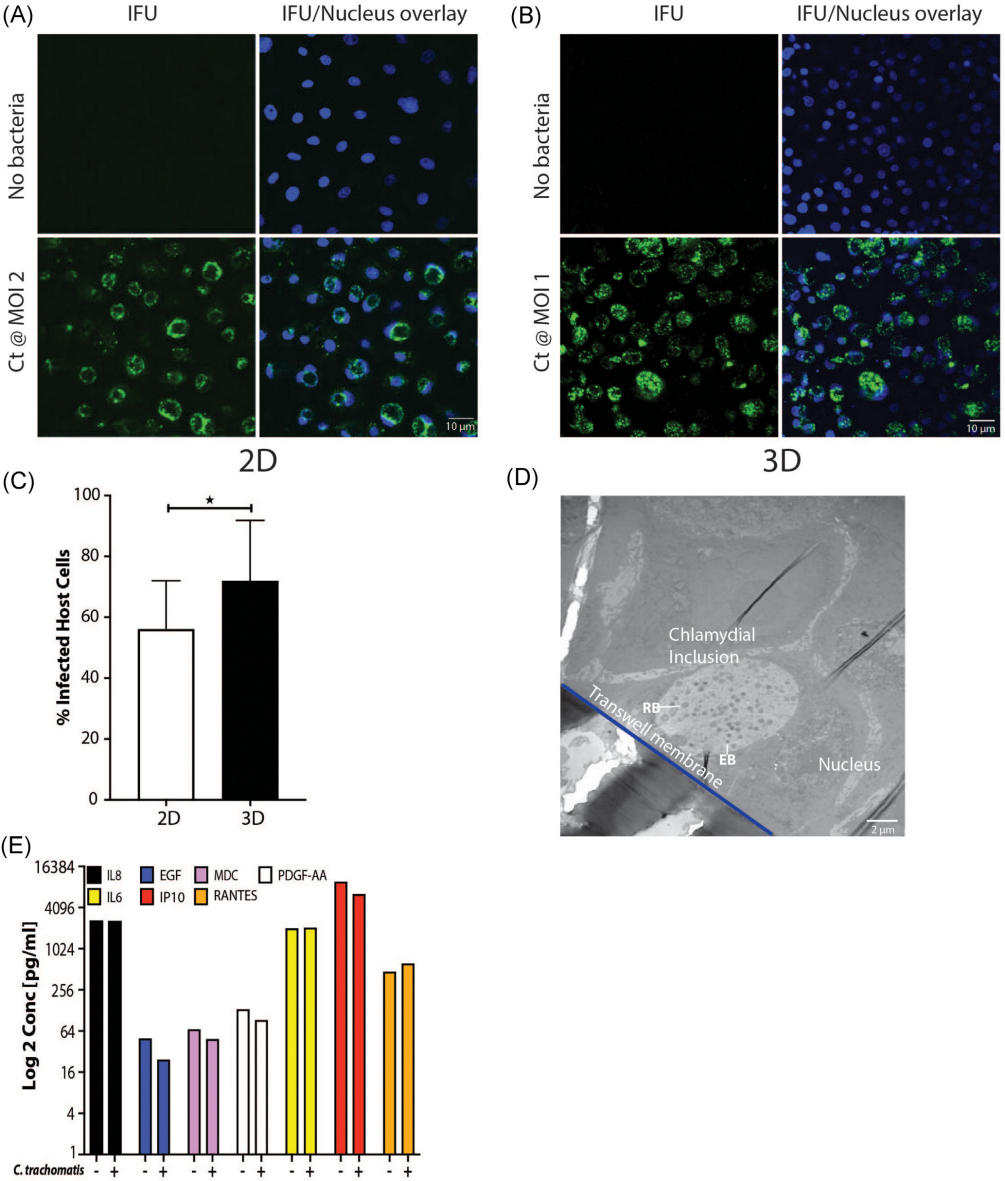
Infection of the 3D cervical model (A2EN) by *C. trachomatis*. Analysis of chlamydial infectivity on the conventional 2D (coverslip) model by fluorescent imaging **(A)** compared to the 3D (transwell) model **(B)**, and resultant enumeration of infected cells **(C)**. TEM image of infected cells on the 3D model (D). Cytokine profile of uninfected as compared to infected 3D cervical cells (E).

The ability of *C. trachomatis* (Ct) serovar L2 to infect cervical A2EN cells was assessed in both a conventional 2D model (Fig. 4A) of cells grown on coverslips and in the 3D transwell model described herein (Fig. 4B). While both models facilitated relatively robust infectivity (Fig. 4C), the A2EN cervical epithelium 3D model accommodated higher infection (71%) compared to the 2D model (57%; *P*-value .019). Both models were infected with 2 × 10^5^ *C. trachomatis* elementary bodies (EBs) representing a MOI of 2 and 1 for the 2D and 3D models, respectively. The MOI was lower for the A2EN cervical epithelium 3D model, demonstrating that a more efficient infection is achieved in this model. The VK2 vaginal epithelium 3D model was also successfully infected with *C. trachomatis* (data not shown); however, as expected, the level of infectivity was low (25%) since *C. trachomatis* predominantly infects cervical epithelial cells. TEM confirmed the infection, visualizing inclusions containing *C. trachomatis* at various developmental stages (Fig. 4D), with both EBs (infectious particles) and reticulate bodies (RBs; metabolic/replicating particles) observed. While A2EN cervical epithelial cells are not robust producers of cytokines (Buckner et al. 2011), we investigated the profiles of some common cytokines and found appreciable levels of IL-6, IL-8, IP10, and RANTES (Fig. 4E). These results are similar to those observed by Buckner et al. (2011), where perceptible levels of IL-6, IL-8, IP10, and RANTES were detected. In addition, we observed that the cytokine response of the model in the presence or absence of a chlamydial infection was comparable to that previously observed in this and other human and mouse cell lines (Buckner et al. 2013, Hwang et al. 2015, Bua et al. 2019, Yang et al. 2019). These results indicate that the A2EN cervical epithelium 3D model could serve as a suitable platform for studies of chlamydial infection.

**Figure 5.**
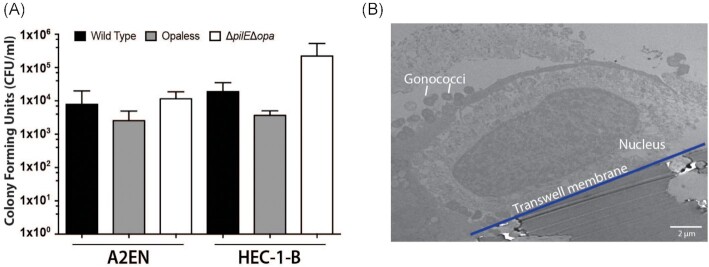
Infection of the 3D cervical cells (A2EN) by *N. gonorrhoeae* (Gc). Transmigration of Gc across the cervical epithelium model is similar to that obtained with a commonly used cell line (HEC-1-B) **(A)**. TEM image of Gc attached to 3D cervical epithelial cells **(B)**.

Another common sexually transmitted pathogen is *N. gonorrhoeae* (Unemo et al. 2019), with anecdotal evidence suggesting that *N. gonorrhoeae* infection might increase the risk of *C. trachomatis* infection (Batteiger et al. 1989, Creighton et al. 2003). Utilizing wildtype and mutants of a common *N. gonorrhoeae* laboratory-adapted strain FA1090, we showed that transmigration of *N. gonorrhoeae* takes place within 6 h in the 3D cervical A2EN model. This is similar to the transmigration period observed with a HEC-1-B 3D cell model (Fig. 5A), a cell line commonly used to analyze *N. gonorrhoeae* infections (Griffiss et al. 1999, Jarvis et al. 1999, Spurbeck and Arvidson 2008, Edwards et al. 2013). Furthermore, TEM imaging shows *N. gonorrhoeae* attached to the surface of the A2EN cells (Fig. 5B), which is the first step in the pathogenic cycle. These results suggest that the 3D A2EN cervical epithelium model can also support investigations of *N. gonorrhoeae* pathogenesis.

### The 3D vaginal model can sustain the growth of vaginal bacteria

**Figure 6.**
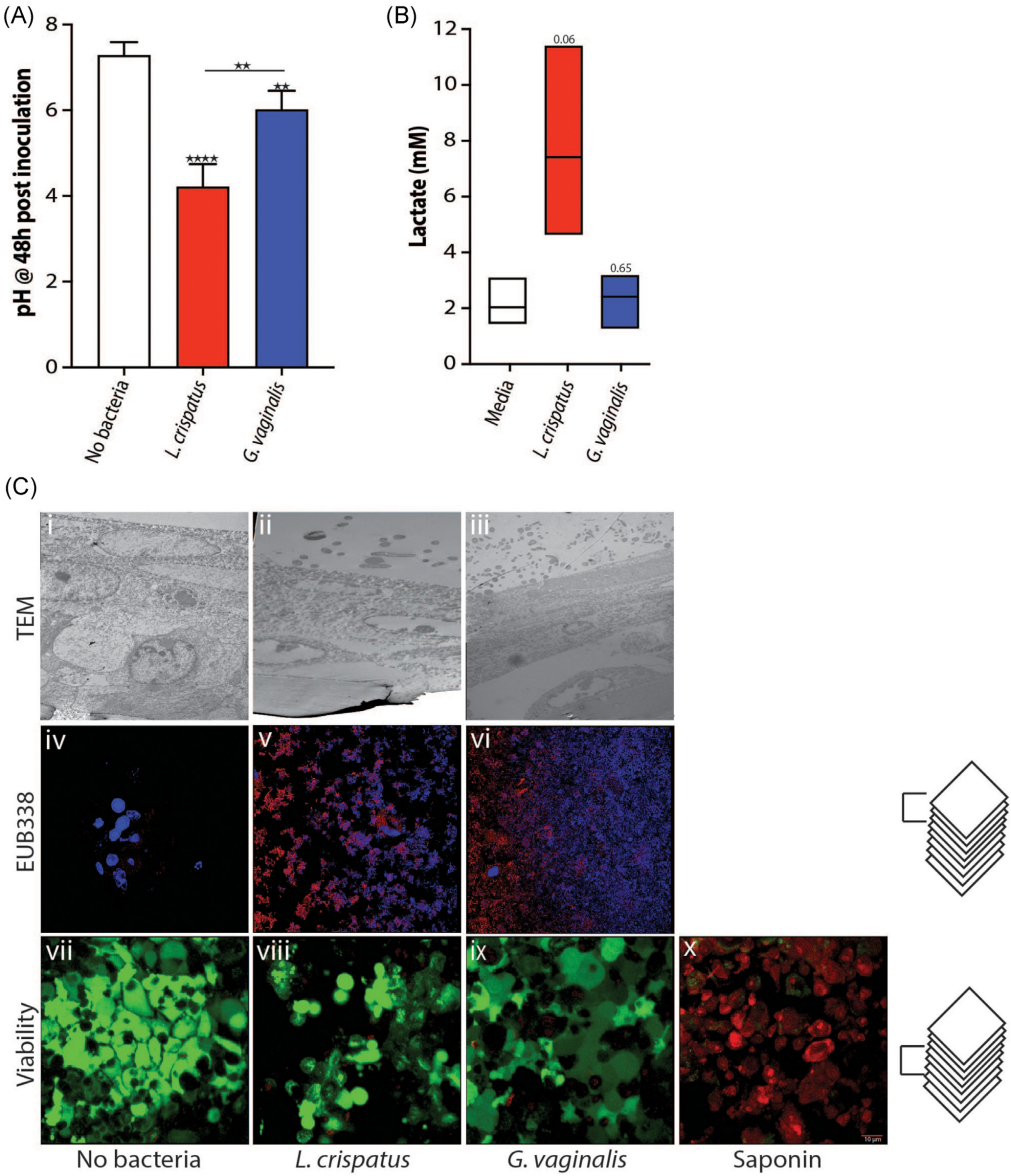
The 3D vaginal (VK2) epithelium model supports the growth of vaginal bacteria. pH **(A)** and D(-) lactate concentrations **(B)** of apical media after 48 h of anaerobic bacterial growth on 3D VK2 cells. TEM, FISH, and viability images of bacteria and host cells after 48 h of growth **(C)**.

The vaginal microbiota plays a vital role in the cervicovaginal microenvironment (Ma et al. 2012). We developed conditions that afford the growth of *L. crispatus* and *G. vaginalis* on the 3D vaginal epithelium model. These two species are prominent members of vaginal bacterial communities found in optimal and nonoptimal conditions, respectively (McKinnon et al. 2019). These bacteria were used to inoculate the 3D vaginal epithelium model and were shown to grow for at least 48 h under anaerobic conditions. Growth was first demonstrated by measuring the pH of the culture medium in the apical compartment after 48 h of growth. Medium containing *L. crispatus* had a significantly lower pH of ∼4.2 as compared to *G. vaginalis* (pH 6.0; Fig. 6A); as expected, *L. crispatus* acidified the microenvironment, while *G. vaginalis* did not. *In vivo* acidification is driven by the production of lactic acid by *L. crispatus*, typified with a higher proportion of D(-) lactate as compared to L(+) lactate (Boskey et al. 2001, O’Hanlon et al. 2013, Witkin et al. 2013). A concentration of 7.41 mM D(-) lactic acid was observed after 48 h of growth with *L. crispatus* compared to 2.42 mM and 2.04 mM with *G. vaginalis* or a no bacteria control, respectively (Fig. 6B). This finding demonstrates that *L. crispatus* is metabolically active and growing in the 3D vaginal epithelium model. Further microscopic analyses using both TEM (Fig. 6C i, ii, and iii) and FISH (Fig. 6C iv, v, and vi) showed the presence of live *L. crispatus* (Fig. 6C ii and v) and *G. vaginalis* (Fig. 6C iii and vi) in the model under anaerobic conditions after 48 h growth. It is important to note that the model was gently rinsed with PBS before fixation; thus any nonadherent bacteria were removed, and only bacteria attached to the epithelial surface or embedded in the mucin matrix were imaged. TEM afforded visualizing the physical localization of the bacteria in close proximity to the epithelial layer, while FISH staining confirmed the robust growth of the bacteria on the model. Viability staining (Fig. 6C vii, viii, and ix) after 48 h of bacterial growth indicated that vaginal epithelial cells remained viable (green staining on Fig. 6C), in contrast to a control comprising of epithelial cells exposed to 1% saponin, which predominantly stain red and indicates dead cells (Fig. 6C).

## Discussion

2D cell culture models have been extensively used to study host–STI pathogen interactions. However, these models lack complexity and do not accurately mimic many of the physiological interactions that occur in host organisms, limiting interpretation and translation to complex human physiology. Previous groups have seeded epithelial cells in transwells and created an ALI by only basally feeding the cells (Kaushic et al. 2011, Lee et al. 2016). Similar systems were developed using rat uterine cells and sometimes incorporated ECMs and hormones (Wira and Rossoll 2003, MacDonald et al. 2007). We have advanced the human models by developing 3D organotypic air–liquid interface models of cervical and vaginal epithelia, including an interstitial collagen compartment and associated fibroblasts. For this model, we used rat tail collagen as the foundation of the ECM simply because while human ECMs are available, their cost is over 3.5 times that of rat tail collagen.

3D epithelial models provide enhanced morphological and physiological cellular structures that can include intercellular interactions (i.e. junctional complexes), complex tissue interactions, and differentiated and polarized epithelial structures, which better mimic *in vivo* cellular behavior (Schmeichel and Bissell 2003, Hjelm et al. 2010, Harrington et al. 2014, Orabi et al. 2017, Xiao et al. 2017, Zhu et al. 2017, Barrila et al. 2018, Ogawa-Tominaga et al. 2018, Gargus et al. 2020). The multilayered structure of these models affords increases in complexity and experimental flexibility, such as the potential addition of different cell types or even immune cells. Hormones are an integral part of the female reproductive tract, and addition of estradiol to our vaginal cell model resulted in enhanced epithelial thickness, demonstrating a physiologically relevant estrogen response of the model. 3D cell culture models partly fill a gap between the cost-effectiveness of 2D cell culture and the complexity and high cost of organoids, organ-on-a-chip systems, or animal models (Dutow et al. 2016, Herbst-Kralovetz et al. 2016, Lavender et al. 2018, Pal et al. 2018, Raterman and Jerse 2019). Animal models can be of limited use to study host–STI pathogen interactions because they often lack anatomical similarity to the human vaginal epithelium. For example, the lower reproductive tract of the mouse, an animal model commonly used in STI research, comprises a keratinized stratified epithelium, while that of humans is not keratinized. The 3D organotypic model we have developed is ideally suited for studies of the pathogenesis of STIs as it replicates many features of human cervicovaginal epithelia without the complexity, experiment-to-experiment variability and/or high cost of organoids and animals. This proposed model will ultimately provide a way to study how the cervicovaginal microbiota interacts with the host and how these interactions increase or reduce the risk of infections by sexually transmitted pathogens.

We have shown that the model supports infection by *C. trachomatis* and *N. gonorrhoeae*, two of the most prevalent infections worldwide. *Chlamydia trachomatis* is an obligate human pathogen that requires host cell internalization for propagation, while *N. gonorrhoeae* can replicate both outside and inside epithelial cells. The 3D cervical model reproduced characteristic features of infection by both pathogens. To show the robust nature of this 3D model, we used the highly infectious *C. trachomatis* serovar L2, which, while a serovar not involved in the most common reproductive tract infections, has been used extensively in research on chlamydial pathogenesis. Previous work (Edwards et al. 2019) has shown that genital serovar D follows the same infection and susceptibility trends as serovar L2 in this model, the only difference being that fewer cells are infected when serovar D is used. One can envision using these models to study coinfections or the role of a primary infection by *C. trachomatis* in susceptibility to infection by *N. gonorrhoeae*, or *vice versa*. Other potential coinfections, including with HSV or HIV, could also be investigated. The model can be enhanced further by adding more complex structures such as endothelial and/or immune cells to the basal compartment.

The ability to grow vaginal bacteria on the 3D models of the vaginal and cervical epithelia is a critical first step toward modeling the *in vivo* complex microenvironment that includes a functional microbiota. Little is known about how the vaginal microbiota contributes to modulating susceptibility to STIs. Previous studies have postulated that indole-producing bacterial species such as *Prevotella, Petpostreptococcus*, or *Peptinophilus* spp. can facilitate *C. trachomatis* replication (Aiyar et al. 2014, Ziklo et al. 2016, 2018), since *C. trachomatis* can use indole to synthesize tryptophan, an essential amino acid that genital *C. trachomatis* strains cannot produce. Tryptophan is present in the host extracellular and cytoplasmic compartments, but can be depleted through the action of interferon (IFN)-γ, which induces tryptophan catabolism by indoleamine-2,3-dioxygenase I (IDO; Shemer and Sarov 1985). Mechanisms such as this have been challenging to study for at least three reasons: (1) it is unethical to perform many of these experiments in humans; (2) there are no cellular or biomimetic models of the cervicovaginal environment that include the microbiota; and (3) key features of the cervicovaginal space such as the dominance of *Lactobacillus* spp. and a low environmental pH (< 4.5) are not found in other mammals that might otherwise be candidate animal models (Stumpf et al. 2013, Yildirim et al. 2014, Miller et al. 2016). The 3D models developed in this study represent the first step toward more advanced models that include complex microbiota. This component is critical, as the cervicovaginal microbiota exists in a mutualistic relationship with the cervicovaginal epithelium and is believed to play an essential role in the risk to STIs. The microbiota is thought to constitute the first line of defense against STIs, but the mechanism(s) by which it exerts its protective effect(s) is/are unknown. Access to a model that reproduces the physiology and microbiology of the cervicovaginal space is, thus critical. We have previously shown that an optimal microbiota dominated by *Lactobacillus* species, such as *L. crispatus*, produces copious amounts of lactic acid and a concomitant low environmental pH (< 4.5). Lactic acid does not directly affect *C. trachomatis* bacteria, but acts on the epithelium by decreasing epithelial cell proliferation, thus significantly inhibiting the infection process (Edwards et al. 2019). On the other hand, microbiota compositions associated with an increased risk to STIs tend to be similar to those observed in association with bacterial vaginosis (BV). BV is a condition, i.e. generally defined by a high pH (> 4.5), a microbiota characterized by the absence of *Lactobacillus* spp. and the presence of an array of strict and facultative anaerobes such as *G. vaginalis, Atopobium vaginae*, and *Prevotella* spp. The mechanisms by which a STI-permissive microbiota increases the risk of infection remains poorly understood. Based on our previous research, we posit that a nonpermissive indigenous microbiota interacts with the cervicovaginal epithelium to establish a homeostatic state that blocks STI and/or reduces disease severity. Conversely, we propose that a permissive microbiota disrupts host epithelial cell homeostasis, allowing STI to progress. Establishing reconstituted STI-permissive or nonpermissive microbiota on an advanced 3D epithelial model will go a long way toward testing these hypotheses and improving our knowledge of the pathogenesis of STIs.

The 3D models developed in this study use relatively inexpensive materials compared to organoids or organ-on-a-chip systems. These low-cost models afford performing replicate experiments in a semi high-throughput set-up. In addition, this 3D model allows performing different analyses from one or replicate transwells, including resistance readings, measurements of pH, metabolite concentrations (i.e. lactate), cytokine concentrations, bacterial enumeration, and imaging (fluorescence, TEM) or omic analysis (DNA/RNA sequencing, proteomics, among others). Lastly, while we developed this system with A2EN cervical and VK2 vaginal cell lines, there is no barrier to using different cell lines more appropriate to the research questions at stake or even from different organ systems or tissues.

## Authors’ contributions

J.R., P.M.B., and V.L.E. designed the research; V.L.E. and E.M. performed the research; V.L.E. and E.M. analyzed the data; V.L.E., J.R., P.M.B., J.P.G., and L.J.F. wrote the paper; and J.R., J.P.G., and P.M.B. obtained the funding.

## Supplementary Material

ftac026_Supplemental_File
