# Supplementary material for: Three-dimensional models of the cervicovaginal epithelia to study host–microbiome interactions and sexually transmitted infections
Source: Pathog Dis. 2022 Aug 4;80(1):ftac026. doi: 10.1093/femspd/ftac026 (PMC9419571; doi:10.1093/femspd/ftac026)
Supplement: ftac026_Supplemental_File [file ftac026_supplemental_file.pdf]

Supplemental Materials

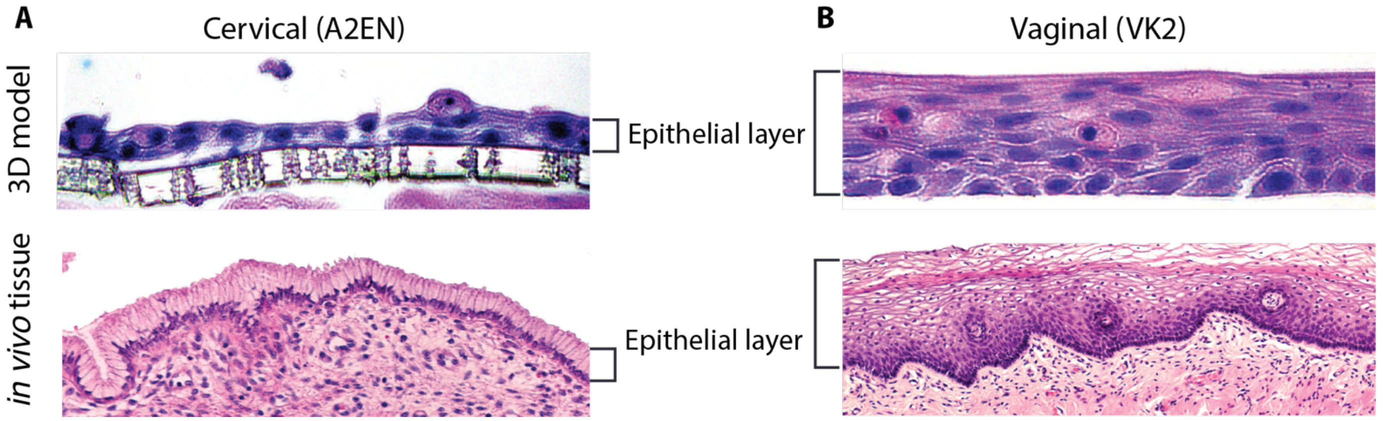

Figure S1. Comparison of the 3D transwell models to *in vivo* tissue images. H&E staining of cervical (A) and vaginal (B) cells from the 3D model compared to in vivo tissue images: Images of *in vivo* tissues were purchased from shutterstock.com.

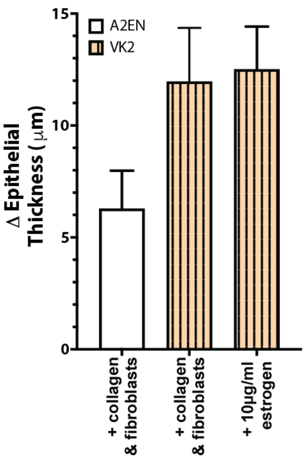

Figure S2. Comparison of the difference in thickness of the epithelial layer of the A2EN and VK2 3D transwell models in the presence and absence of basal collagen and fibroblasts as well as the VK2 3D transwell model when 10ng/ml of estrogen is added to the media for the final 4 days of development.

List of materials, suppliers and catalog numbers

Transwell inserts (Corning #3472)

12 well plates (Corning #3513)

9" Glass pipettes (Corning #7095D)

Countess Automated Cell Counter (Invitrogen #C10227)

Mouse anti-human chlamydia LPS (US Biological #C4250-51F)

Goat anti-mouse Alexa Fluor 488 (Invitrogen #A-11029)

Rabbit anti-human MUC5B (Invitrogen #PA5-82342)

Goat anti-rabbit Alexa Fluor 488 (Invitrogen #A-11034)

Hoechst (Invitrogen #H3570)

FISH probe - EUB338-ATTO [5'-/565/GCT GCC TCC CGT AGG AGT-3'] (Invitrogen)

Histology fixative – 10% formalin solution (Sigma #HT501128)

D/L Lactic acid assay kit (R-Biopharm #11 112 821 035)

Rat tail collagen – 100mg (Corning #354236)

10X RPMI media (Sigma #R1145)

Sterile tissue culture water (Cellgro #25-055-CM)

1M NaOH – sterile filtered (Sigma #S5881)

0.25% Trypsin (Gibco #25200-056)

BJ human fibroblasts (ATCC #CRL 2522)

BJ complete media - DMEM media (Cellgro #15-013-CV) supplemented with 10% FBS (Sigma #F4135)

A2EN cervical epithelial cells (kindly provided by Dr. Allsion Quayle (Buckner*, et al.* 2011))

A2EN complete media - EpiLife media (Gibco #MEPICFPRF) with 100X EDGS supplement (Gibco #S-012-5) and 100X L glutamine (Lonza #17-605E)

VK2/E6/E7 human vaginal epithelial cells (ATCC #CRL 2616)

VK2 complete media - Keratinoctye-SFM (with BPE and EGF) (Gibco 310725-018) supplemented with Calcium chloride (Amresco #E506) and 100X L glutamine (Lonza #17-605E)

HeLa cervical epithelial cells (ATCC #CCL2)

HeLa complete media - Dulbecco's modified Eagle's medium (Corning #15-013-CV) supplemented with 10% FBS (Sigma #F4135)

HEC-1-B endometrial cells (ATCC #HTB-113)

HEC-1-B complete media – MEM alpha (1X) + GlutMaAX (GIBCO #32561-037) supplemented with 10% FBS (Sigma #F4135)

Chlamydia trachomatis serovar LGV II strain 434 (ATCC #VR 902B)

Neisseria gonorrhoeae strain FA1090 – wildtype and isogenic mutants (kindly provided by Dr. Alison Criss (Ball and Criss 2013; Smirnov*, et al.* 2014)

Lactobacillus crispatus (ATCC #33197)

Lactobacillus iners (ATCC #55195)

Gardnerella vaginalis (ATCC #14018)

Difco GC medium base (BD #228920) with 1% Kelloggs supplement prepared as per White and Kellogg (White and Kellogg 1965).

Bacteriological agar (Amresco #J637)

NYCIII medium: 10 g/L proteose peptone, 10 g/l beef extract, 5 g/l yeast extract, 5 g/L NaCl, 1.2 g/L $MgSO_4$, 2 g/L $MnSO_4.H_2O$, 5.7 g/L K_2HPO_4, 20 g/L glucose, 10% FBS.

Tryptic Soy Broth (Fluka #T8907) supplemented with 5% Horse serum (GIBCO #26050-088)

β-Estradiol (Sigma E4389)

Supplemental References

Ball LM, Criss AK. Constitutively Opa-expressing and Opa-deficient *Neisseria gonorrhoeae* strains differentially stimulate and survive exposure to human neutrophils. *Journal of bacteriology* 2013;**195**: 2982-90.

Buckner LR, Schust DJ, Ding J *et al.* Innate immune mediator profiles and their regulation in a novel polarized immortalized epithelial cell model derived from human endocervix. *Journal of reproductive immunology* 2011;**92**: 8-20.

Smirnov A, Daily KP, Criss AK. Assembly of NADPH oxidase in human neutrophils is modulated by the opacity-associated protein expression State of *Neisseria gonorrhoeae*. *Infect Immun* 2014;**82**: 1036-44.

White LA, Kellogg DS, Jr. *Neisseria Gonorrhoeae* Identification in Direct Smears by a Fluorescent Antibody-Counterstain Method. *Appl Microbiol* 1965;**13**: 171-4.
